# Ohio State Dental Society

**Published:** 1867-07

**Authors:** 


### OHIO STATE DENTAL SOCIETY.

The late meeting of the Ohio State Dental Society was one of the most profitable and pleasant meetings we have lately attended. Not as many were present as we had a right to expect. The warm weather, the stringency of the money market, the busy season of the year, the feeling that it would be pleasanter to attend the "American Dental Association," so soon to meet in our bounds, all had something to do in keeping members away, but not half so much as a want of interest in the progress of the profession, and a confirmed habit of neglecting to contribute, even sympathy, to promote this progress.

But a goodly number of the wide-awake members were on hand. There not being a quorum in the morning, no formal business could be done; but the time was spent in a pleasant and profitable exchange of sentiments and opinions, and at half past one, the society came to order, and went, at once, to business with an energy that soon made up for lost time. No report of the discussions was kept. (Those who expect to be blessed by such meetings had best be on hand.)

We have not the time, space, or disposition to give a full detail of the many good points of the meeting.

Under the head of "Preparations of gold for filling teeth," the subject of "crystal gold" was somewhat fully ventilated, as well as the peculiar properties of "shred gold."

"Of new appliances and apparatus," Dr. Dunn, of Delaware, Ohio, exhibited his new patented method of making porcelain work. The improvements on the old style, or "Loomis' patent" are radical and valuable. We expect to notice this at more length, hereafter.

Under the same head, Dr. Berry, on his own behalf, and that of others associated with him, exhibited specimens of Aluminum plate, stating that the details of the process of mounting artificial teeth on pure aluminum are not all perfected, but that it was believed, by the experimenters, that the difficulties are all overcome, and that soon this metal will be in general use, both for partial and full sets. The history of these experiments is about this: When the late Jno. T. Toland opened his Dental depot, we employed him to obtain for us a supply of aluminum, for experiment in reference to its application to mechanical dentistry. He succeeded in getting it by the March or April following. Our first experiments had reference to the properties of the metal, chemically considered. These proved satisfactory. A few mechanical manipulations were *not* satisfactory. A failure of health occurred, and all mental effort that could be abandoned was laid aside. At Boston, last summer, we procured a fresh supply of aluminum, resumed experiments, were again prostrated, and, on getting up again, made arrangements with Drs. Berry, Drake, and Williams to co-operate, and so we have been experimenting ever since. We believe that the success will reward the toil, and that the profession and the public will both be blessed by the substitution of a noble metal for the objectionable rubber. With Dr. Dunn's improved porcelain for full sets, and aluminum for both full and partial pieces, the usurper, Vulcanite is likely soon to meet the fate of its prototype, Maximilian; and in JOSIAH AND Co. it will have as sincere mourners, if not as disinterested, as the latter has in the crowned heads of Europe.

We are glad to know that others are experimenting with aluminum. The more attention devoted to it, the more likely will good results follow.

At the evening session of the second day, some time was devoted to a question of ethics. A resolution was adopted condemning the employment of agents, by dentists, to go from house to house, to solicit and perform operations. We regard the point as fully met by the code of ethics, but it is no harm to have "line upon line."

At a late hour, the society adjourned till its annual meeting in December. W.

